# Institutional Notes and News

**Published:** 1909-09-11

**Authors:** 


### Institutional Notes and News.

### NEW MANAGER OF THE LEEDS INFIRMARY.

The Leeds Infirmary Board has appointed Mr. Fred J. Bray, of Leicester, to be the general manager of the Infirmary in succession to the late Mr. Thomas Blair. Mr. Bray has held the post of assistant house governor and secretary of the Leicester Infirmary for the past four years. There were 73 applications for the position, three of whom were selected by the House Committee and brought before the Board. The voting was ultimately in favour of Mr. Bray, who was elected. Mr. Bray has taken great interest in the Workpeople's Hospital Fund in Leicester, and it is claimed that in proportion to population Leicester has the largest collection from the workpeople in all England. A sum of no less than £12,000 is collected from the workshops. Mr. Bray will endeavour to take over his new duties not later than October 1.

### A NEW CONSTRUCTION MANUAL.

Messrs. William Hodge and Co., Edinburgh and Glasgow, have in the press and will shortly publish a work entitled *Construction, Equipment, and Management of a General Hospital*, by Donald J. Mackintosh, M.B., M.V.O., Medical Superintendent of the Western Infirmary, Glasgow. This comprehensive manual should be of the greatest value to medical men engaged in hospital work, or who may think of selecting hospital administration as a career. It will probably be of even greater value to architects charged with the construction or alteration of hospitals. Dr. Mackintosh, as is well known, has made a special study of the theory and practice of hospital construction and administration, and this book may be accepted as the latest pronouncement on this important and difficult subject. Besides plans of the most approved types of wards and auxiliaries the book contains over 60 carefully reproduced illustrations showing, *inter alia*, wards, operating theatres, laundry and special departments, all kinds of hospital furniture and requirements, etc. It it anticipated that this book will become a manual for daily use among all public authorities that have the care of hospitals. The price of the book is 10s. 6d. net.

### WESTMINSTER HOSPITAL.

The governors have obtained a temporary injunction against the trustees of the Wesleyan Twentieth Century Fund. This injunction prevents the building of the Memorial Hall above the height of the old Aquarium until the result of the trial is known. It was pointed out that if the building were erected the light of many of the wards would be considerably interfered with, and the recovery of the patients thereby retarded. Anyone who has seen the models, erected to scale, can observe at a glance that the hospital is quite dwarfed, and the street between the two being so narrow, practically no light could enter the hospital on that side. What the result would be it is impossible to say, but that artificial light would be necessary in the daytime is certain, and no amount of compensation could compensate for the loss of light and air. It is hoped that the trial will be brought off as speedily as possible. By the will of the late Mr. Gorringe the hospital benefits to the extent of £50,000. The legacy is a portion of £400,000 left in trust for Mrs. Gorringe, which at her death is to be divided between eight charitable institutions.

### THE HOPE HOSPITAL.

The British Medical Association has advised its members not to apply for the position of resident medical officer at the Hope Hospital, Salford, and the recommendation so far has been followed. Dr. J. H. Taylor, hon. secretary of the Salford branch of the Medical Association, alleges that the general treatment of the medical men at the hospital had been intolerable, and that no doctor would go there as a resident officer "until the Guardians altered the whole of their conduct towards them." There are several grievances, but they seem to centre in this, that the Guardians, or some of them, are too meddlesome. "The medical men," Dr. Taylor told a representative of the *Pendleton Reporter*, "can do nothing right for the Guardians, who are constantly nagging." Mr. J. Bratherton, chairman of the Hospital Committee, told a newspaper representative that the action of the British Medical Association is placing the Guardians in an unfair position before the ratepayers. He repudiated the suggestion which has been made that there is any animus on the part of the Guardians against the retiring medical officer. "I deny entirely," he said, "that we are actuated by any personal feeling in resolving not to renew the appointment. With other members of the board I am now assisting the doctor's candidature for another appointment." Mr. Bratherton would not admit that the hospital was under-staffed, nor that the salary was too low. "When a shortage of staff was pointed out to us by the Local Government Board, the Guardians," he added, "remedied it immediately, and only at the last Committee meeting Mr. J. Lowry, the Local Government Board inspector, congratulated the members on the improvements that had been made. A month ago the Guardians agreed on an expenditure of nearly £3,000 for the better equipment of the operating theatre and the administrative parts of the institution."

### NEW MENTAL HOSPITAL AT TOXTETH.

The Toxteth Board of Guardians have just added to the Smithdown Road Workhouse two important blocks which are to serve as mental hospitals for 200 males and 100 females. Each hospital is two stories in height, and of neat and substantial design. The complete cost of both hospitals, including furnishing, fire mains, hydrants, and heating is about £20,000, or equal to £70 per bed. These are considered the cheapest hospitals of the kind built, taking into consideration the stability and the up-to-date sanitary fittings. The architect is Mr. Walter W. Thomas.

### THE LATE REV. DR. DAWSON BURNS.

At the board meeting of the London Temperance Hospital, held on Tuesday, August 24th, the following resolution was moved by the Chairman, Sir T. Vezey Strong :—"That it is with a feeling of profound sorrow, and a sense of great and irreparable loss, we, the members of the board of management of the London Temperance Hospital, record the death, on Sunday, the 22nd of August, 1909, in his eighty-first year, of the Rev. Dawson Burns, the Honorary Secretary, our beloved friend and colleague. We recall with gratitude his long and devoted service to this institution, which, commencing on February 17, 1871, when he accepted the arduous post of honorary secretary of the provisional committee, continued in never-failing generosity until the day of his death, he taking an active part in the proceedings of the last board meeting on the previous Tuesday. The quality of his service was always of the best, ever enriched by his scholarly attainments, shrewd foresight, sound judgment, untiring energy, and lovable personality. His removal by death involves the Hospital in a loss well-nigh inexpressible, and we, his friends, in a deep and sorrowful bereavement: a bereavement the pain of which is only mitigated by a knowledge of his short and painless illness, a realisation of his long, useful and devoted life, and the happy memory of many years of entirely felicitous comradeship. May it be ours to follow humbly in his footsteps, to strive to carry on the work of this Hospital, of which he was one of the principal founders, and in the establishing of which he devoted so many years of able and faithful service, and, generally, to further the cause of Temperance, to which he so unreservedly dedicated his life. To the members of his family we respectfully tender the expression of our heartfelt sympathy."

### THE WORTHING HOSPITAL.

The annual meeting of this hospital was held on August 27, Colonel A. Henty, J.P., presiding. Mr. Timms, in moving the adoption of the report, said the increase in the number of patients pointed to the necessity for the recent addition to the medical staff, and the institution would benefit by the appointment of four more medical officers. It was necessary that the public should realise that the number of patients increased every year, and that the expenses must go up in proportion. Subscribers should do their best to bring the institution to the notice of those who were not subscribers, and point out its benefits. Mr. F. W. Wink, in seconding, remarked that it was very satisfactory to find that the hospital was so generously supported by the employees of various firms in the town. Dr. Hinds referred to the proposal to provide a permanent memorial of the late Mr. A. H. Collet—for many years a member of the hon. medical staff. Mr. Collet had devoted a great deal of his time and energy to the institution, and it seemed very desirable that a suitable memorial should be erected. A new out-patients' department had been suggested, and this would be a most useful and fitting memorial, but the whole question was one of funds. The cost of the proposed out-patients' department had been spoken of as about £2,000. This might be somewhat in excess of the sum required, but at any rate the cost would be considerable, and it was hoped the public would respond generously to the appeal. Commenting on the statement of accounts, the Chairman said he thought the amount of the annual subscriptions was very small for such a charitable town as Worthing. The revenue from this source for the past few years had been £584, £576, £581, and, during the past year, £596. There was an improvement last year, and having regard to the sum raised in addition for the purchase of land, the support received was fairly satisfactory; but large sums were contributed to other objects, and in many cases sent away from the town, and he would like to see the amount subscribed to the local hospital increased by quite £1,000. At present the committee had to cut things very fine, and it was regrettable in a town of that size that this should be the position.

